# Healthy Bus Drivers, Sustainable Public Transport: A Three-Time Repeated Cross-Sectional Study in Switzerland

**DOI:** 10.3389/ijph.2023.1605925

**Published:** 2023-06-21

**Authors:** Viviane Fiona Mathilde Remy, Irina Guseva Canu

**Affiliations:** Center for Primary Care and Public Health (Unisanté), University of Lausanne, Epalinges-Lausanne, Switzerland

**Keywords:** mental disorders, working conditions, musculoskeletal disorders, SARS-CoV-2 pandemic, driving accidents

## Abstract

**Objectives:** To study the change in the prevalence of bus drivers’ health outcomes between 2010 and 2022 and their relationship with working conditions.

**Method:** Unionized bus drivers completed a self-administered questionnaire in 2010, 2018, and 2022 on 13 health outcomes, sick leaves, and accidents and working conditions and their change during SARS-CoV-2 crisis. For outcomes which prevalence increased since 2010, we performed logistic regression models adjusted for covariates.

**Results:** The study sample included 772 participants in 2010, 393 in 2018, and 916 in 2022. The most prevalent health problem (≥50%) was shoulder or neck muscle pain. The most tedious working conditions were working days over 10 h. Shoulder or neck pain, sleep disorders, sick leaves, and accidents increased since 2010 and were associated with working conditions, and co-morbidity. The SARS-CoV-2 pandemic had additional negative consequences.

**Conclusion:** Most bus drivers’ working and health conditions worsened in the last 12 years. Given the study design, the results deserve a cautious interpretation and generalization. Cohort studies should confirm these results and inform interventions targeting the most tedious and harmful working conditions.

## Introduction

Today, the sustainability of a public transport company is mainly referred to as its financial sustainability. This neglects one important factor: the workers. Note, bus drivers (BDs) are considered as one of the most diseased occupational groups worldwide [1–3]. This is a problem for public transport sustainability as in the past decade the workforce shortage became a salient concern in many countries [4–11]. This situation forces companies to reduce their offer or even cease their activity [4, 6, 10]. The reasons for the bus driver shortage are well-known: a relatively low salary and demanding working conditions [9, 11]. Although the latter can vary from country to country and from one company to another, they have been depicted as tedious for many years [3, 12–14]. Indeed, bus drivers are exposed to numerous occupational and environmental hazards at their workplace. This includes the organizational hazards such as long and irregular working hours, work during the weekends, night work, and split shifts [2, 14, 15] and physical demands with repetitive movements, awkward seating for a long period, and assisting passengers with disabilities [2, 14, 15]. The stressful working environment depending on driving but also weather conditions is another category [14, 15], closely related to the safety concerns such as accidents and altercations with other road users and passengers [2, 14, 15]. Moreover, several physical, chemical, and biological hazards are present in the BDs’ work environment leading to exposure to noise [15–19], vibration [2, 15, 18–21], air pollution [2, 15, 22, 23], fungi and, since 2020, SARS-CoV-2 [24–26].

**FIGURE 1:**
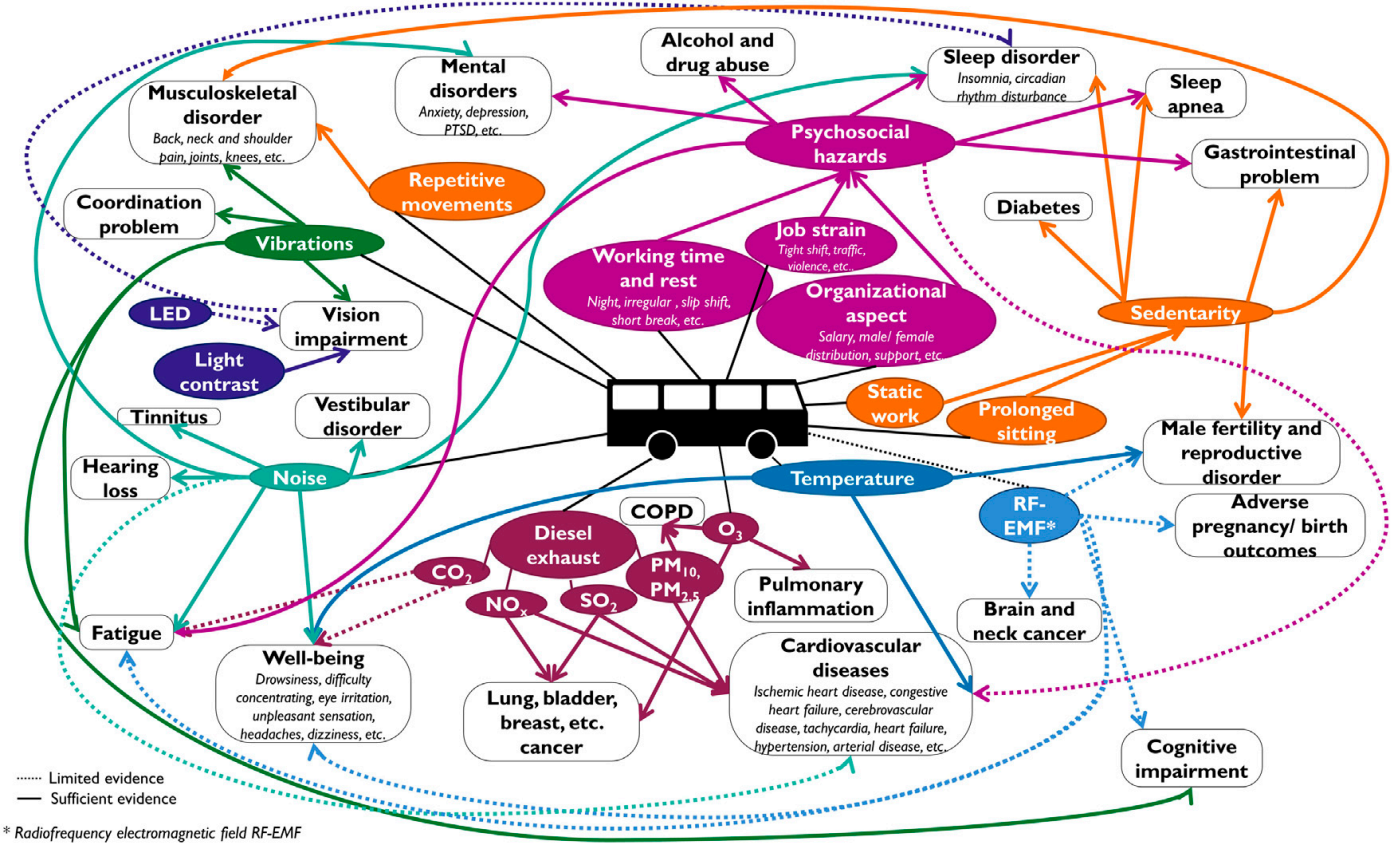
Mind map of the bus drivers’ exposure and potentially related health problems, based on the litterature review (Stratégie Energétique et Santé, Switzerland, 2022).

These occupational exposures could promote a variety of diseases, including lung cancer [22, 23, 27, 28] and other respiratory diseases [15, 23, 29], cardiovascular diseases [2, 14, 15, 18, 23, 30, 31], gastrointestinal diseases [2, 14, 15, 21, 23, 28, 29], hearing loss [15, 16], musculoskeletal disorders [2, 14, 15, 18, 20, 29, 32, 33], mental health issues [2, 12, 14, 15, 30, 34, 35], sleep disorders and fatigue [2, 12, 14, 15, 17, 29]. Figure 1 summarizes the BDs’ exposures and potentially associated health problems.

In Switzerland, compared to the general population, BDs have been identified as at higher risk of mortality from lung cancer [27, 28], gastrointestinal cancer [28], and suicide [36]. No data on occupational exposures were available to investigate the potential causes of this excess mortality. However, it was possible to disentangle occupational risk factors from socio-economic and environmental factors and to confirm the role of occupational risk factors independently from the others in lung cancer mortality [37] and suicide [37, 38]. Regarding the suicide risk factors, a high prevalence of mental and behavioral disorders, substance-related and addictive disorders, and mood disorders were reported among Swiss transport workers [39].

Yet, little is known about the exposure and health status of Swiss BDs specifically. The only information comes from surveys conducted by a trade union active in the transport field [40, 41], but this information remains unpublished and consequently, cannot reach the scientific community and stakeholders. This study aims to fulfill this gap and make use of available and newly collected scientific data to describe the work and health conditions of Swiss BDs with a focus on the most prevalent health problems.

## Methods

### Study Design

This repeated cross-sectional study was based on data collected at three-time points: 2010, 2018, and 2022. The 2010 survey was initiated by one of the three unions representing BDs in Switzerland and was particularly active concerning their health and working conditions [40]. The questionnaire developed for this survey was based on their bus driving experience and addressed the topics such as ergonomics, and difficulties at work but also its positive aspects. In 2018, the union conducted a second survey, using the same self-administered questionnaire to compare the reported proportions of exposed and diseased BDs with those found in the first survey [41]. In 2022, the present study was authorized by the Swiss ethics committee, enabling us to make use of the previously collected data and to conduct a new survey in collaboration with all Swiss unions active in public road transport. All surveys were anonymous and used a proper distribution channel. Consequently, we expect no or a very little overlap between the three study samples and consider the study samples as independent. This allows analyzing the change in the prevalence of health outcomes and exposures between different time points (i.e., 2022 *versus* 2010; 2022 *versus* 2018; 2018 *versus* 2010).

### Participants and Recruitment Method

In 2010 and 2018, the source population included BDs affiliated with union 1. This union covers only a part of Switzerland and a third of the unionized Swiss BDs. In 2022, the source population was extended to the BDs affiliated with any of the three unions, covering the entire Swiss territory. The inclusion criteria were similar in all the surveys; the study sample included active BDs aged between 18 and 67 years old. The upper age limit corresponds to 2 years after the retirement age, as some Swiss BDs continue to work after retirement.

The 2010 and 2018 surveys were distributed and collected on paper by union 1 representatives in each bus company with affiliated BDs. The surveys took place between the spring and fall of the corresponding year. The 2022 survey was a multilingual online questionnaire created in RedCap [42, 43] and distributed by email to 4,324 potentially eligible participants by their unions. This survey took place between 22 February and 6 April, 2022. Before the survey, the unions organized a joint survey promotion campaign [44]. Moreover, the information regarding the survey with a link and a QR code to the survey was published in the monthly newsletter of the three unions and distributed to all union members.

The participants were not paid and did not receive any kind of compensation. The survey responses were anonymous. The study protocol was submitted to the competent ethics committee, which waived the need for informed written consent (CER-VD decision number 2021-01089).

### Data Collected

We used a multilingual questionnaire encompassing four parts (Supplementary File S1).

#### Demographic and Socio-Professional Characteristics

This part included questions on sex, age, education attainment, apprenticeship, and county of residence. The company, seniority within the company, working rates, and employment as BD in other companies were also addressed.

#### Health Problems and Driving Accidents

BDs were asked to check off all health problems experienced at least once a month. The 13 proposed health problems were abnormal fatigue, shoulder or neck muscle pain, upper limb muscle pain, lower limb muscle pain, back pain, headaches, stomach pain, stress, anxiety, irritability, sleep disorders, appetite, or digestion problems, and hypersudation. Drivers were also asked to answer with yes or no to the following questions: “Have you had to miss work for health reasons in the past year?” coded as sick leave, “Have you had a work-related accident with time off work in 2021?” coded as an accident, and “Do you always drive while in full control?” coded as driving in unfit conditions. If the answer was “no” to the last question, we asked to indicate the reasons.

#### Working Conditions

BDs were asked to grade twelve working conditions on a scale from 1-not tedious to 4-very tedious. The work conditions proposed were night work (from 10 p.m.), evening work (from 6 p.m.), Sunday work, difficult traffic conditions, driving time over 4 hours, a working day over 10 h, delayed traffic, aggressive customers, aggressiveness of other road users, traffic disruptions (accidents/deliverymen parking), cyclists’ unfair behavior, and long periods without access to toilets.

#### SARS-CoV-2

The fourth part concerned SARS-CoV-2 management and its consequences on health and working conditions. These data are described in details elsewhere [45].

### Data Management and Statistical Analysis

The database with completed questionnaires was downloaded from RedCAP software and examined for completeness and outliers.

Descriptive statistics, mean and standard deviation [M ± SD], and percentage [*n*, %] were computed to describe the socio-professional and demographic characteristics, health problems, and work tediousness in the study sample at each of the three-time points. Furthermore, a comparison between the sample and target population was made based on the demographic informations from Federal Statistical Office.

To assess the change in health problem prevalence across three surveys conducted during the last 12 years, we performed multivariate logistic regression analyses adjusted for sex, age, seniority, and residence regions using the samples from the three time points. This analysis was restricted to BDs affiliated with union 1. For the health problems, which prevalence has significantly increased in 2022, we performed analyses to assess the relationship with BDs’ socio-professional and demographic characteristics, comorbidity, work tediousness, and SARS-CoV-2 crisis management and impacts. This analysis was conducted using the 2022 survey data and included all responding BDs regardless of their union. Furthermore, for each outcome, we constructed multivariate logistic regression models based on the direct acyclic graph (DAG) [46] (Supplementary File S2).

Odd ratios were reported with a 95% confidence interval. The significance level was set at 0.05 and the *p*-values were two-tailed. Data cleaning and descriptive analysis were performed with R version 1.3.1093 [47]. Logistic regression analyses were conducted using Stata, version 17 [48].

## Results

### Comparison Between the Sample and the Target Population

The comparison of our sample to the target population showed similar distribution per age group and sex (*p*-value >0.05, chi-square test), though an over-representation of Western Switzerland and Ticino was observed (68.6% in our sample *versus* 48% Swiss BDs in Western Switzerland and Ticino in the target population). Conversely, the German-speaking regions were under-represented (23% in our sample *versus* 52% of Swiss BDs).

### Health Problems, Accidents, and Tedious Working Conditions in 2022 Survey

**FIGURE 2:**
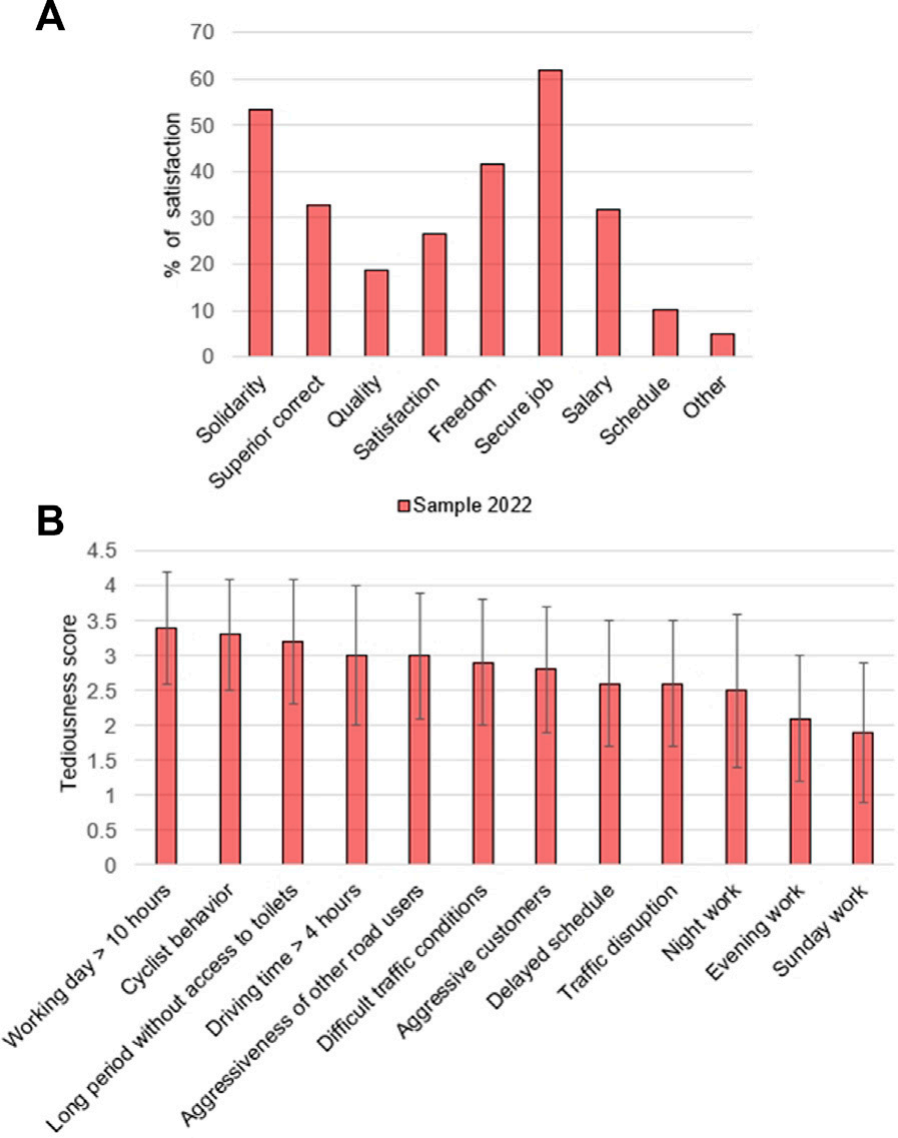
**(A)** Appraisal of positive aspects in work conditions **(B)** Appraisal of tediousness in work conditions among Swiss unionized bus drivers (2022 survey) (Stratégie Energétique et Santé, Switzerland, 2022).

**FIGURE 3:**
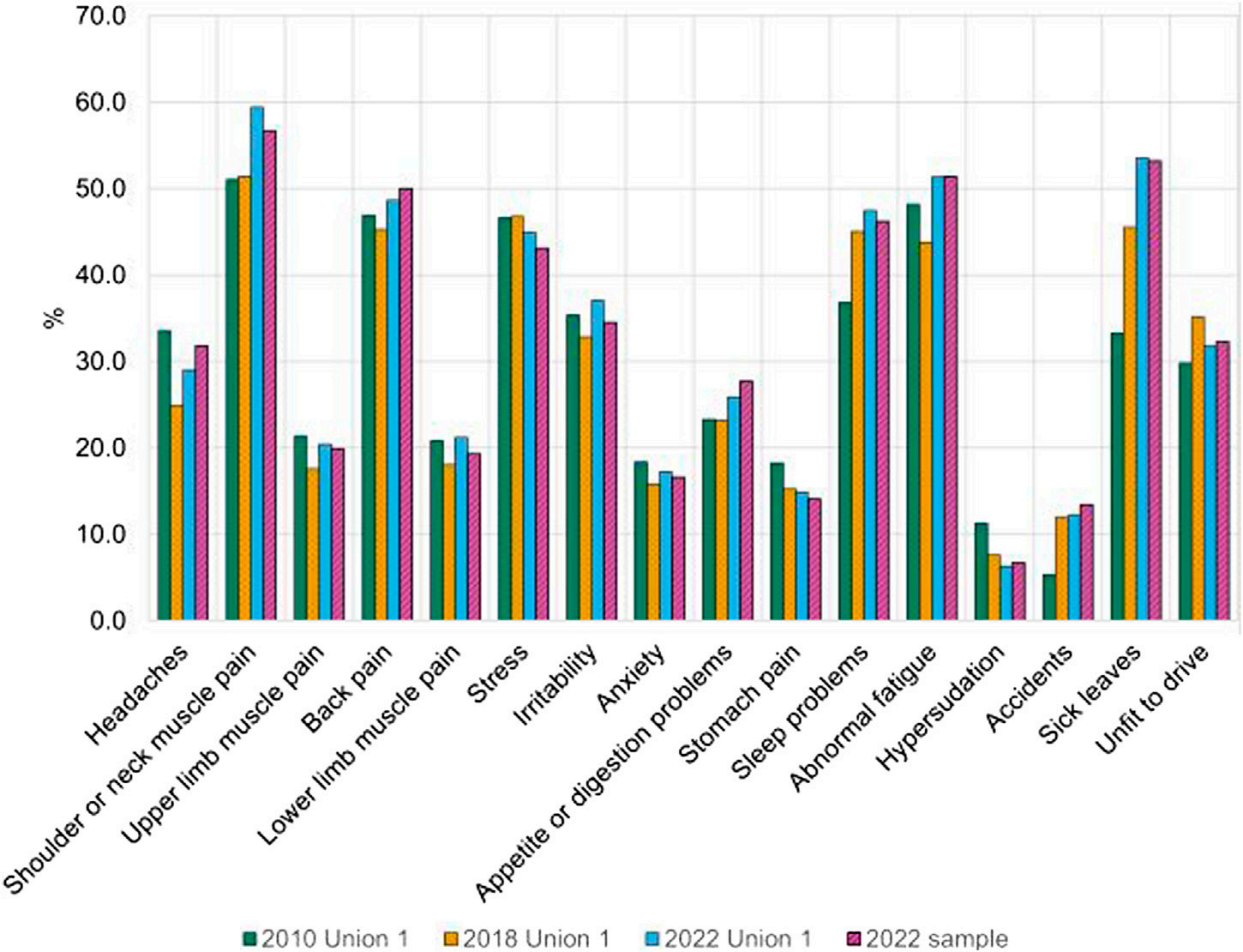
The prevalence of health problems and driving accidents for the three-time points among unionized bus drivers (Stratégie Energétique et Santé, Switzerland, 2022).

The response rate of the 2022 questionnaire was 21.2% (916 participants). The socio-demographic characteristics of the complete 2022 sample are presented in Table 1. The distribution of study participants by unions is represented in Supplementary File S3. The prevalence of health problems, accidents, and the scores of work tediousness are summarized in Figures 2, 3. The three most prevalent health problems were shoulder or neck muscle pain (57.4%), abnormal fatigue (51.1%), and back pain (49.7%). Furthermore, the prevalence of sick leave in 2021 was 53.2%. Almost a third of drivers (31.4%) drove in an unfit condition in 2021, the three main cited reasons being fatigue, muscular pains, and pressure from the companies or colleagues. The three most tedious working conditions were working days over 10 h (3.4, SD = 0.8), cyclists’ behavior (3.3, SD = 0.8), and long periods without access to toilets (3.2, SD = 0.9). The three most valued working advantages were the security of the job (62.4%), solidarity between colleagues (54.7%), and freedom (42.3%).

**TABLE 1:** Socio-professional and demographic characteristics per union of all samples (2010 sample, 2018 sample, and 2022 sample) (Stratégie Energétique et Santé, Switzerland, 2022).

	2010		2018		2022									
	Union 1		Union 1		Union 1		Union 2		Union 3		None		Total	
	*N*	%	*N*	%	*N*	%	*N*	%	*N*	%	*N*	%	*N*	%
Total	772	100	393	100	511	100	187	100	151	100	67	100	916	100
Gender														
Male	715	92.6	343	87.3	439	85.9	168	89.8	113	74.8	52	77.6	772	84.3
Female	36	4.7	32	8.1	70	13.7	17	9.1	36	23.8	6	9.0	129	14.1
Missing	21	2.7	18	4.6	2	0.4	2	1.1	2	1.3	9	13.4	15	1.6
Age group														
<35 ans	104	13.5	60	15.3	75	14.7	23	12.3	20	13.2	7	10.4	125	13.6
36-45 ans	220	28.5	97	24.7	121	23.7	31	16.6	36	23.8	11	16.4	199	21.7
46-55 ans	296	38.3	134	34.1	178	34.8	57	30.5	51	33.8	21	31.3	307	33.5
>56 ans	118	15.3	89	22.6	127	24.9	69	36.9	41	27.2	16	23.9	253	27.6
Missing	21	2.7	13	3.3	10	2.0	7	3.7	3	2.0	12	17.9	32	3.5
Region														
Espace Mitteland	282	36.5	101	25.7	169	33.1	68	36.4	33	21.9	28	41.8	298	32.5
Northwestern Switzerland	50	6.5	3	0.8	30	5.9	9	4.8	24	15.9	0	0.0	63	6.9
Eastern Switzerland	48	6.2	4	1.0	19	3.7	12	6.4	28	18.5	1	1.5	60	6.6
Lake Geneva Region	215	27.8	164	41.7	188	36.8	33	17.6	0	0.0	7	10.4	228	24.9
Ticino	58	7.5	51	13.0	56	11.0	41	21.9	0	0.0	7	10.4	104	11.4
Central Switzerland	99	12.8	7	1.8	30	5.9	5	2.7	50	33.1	0	0.0	85	9.3
Zurich	0	0.0	0	0.0	19	3.7	19	10.2	16	10.6	6	9.0	60	6.6
Missing	20	2.6	63	16.0	0	0.0	0	0.0	0	0.0	18	26.9	18	2.0
Education level														
Mandatory	326	42.2	141	35.9	194	38.0	81	43.3	74	49.0	24	35.8	373	40.7
Secondary	283	36.7	135	34.4	192	37.6	48	25.7	55	36.4	19	28.4	314	34.3
University	153	19.8	84	21.4	121	23.7	56	29.9	20	13.2	12	17.9	209	22.8
Missing	10	1.3	37	9.4	4	0.8	2	1.1	2	1.3	12	17.9	20	2.2
Apprenticeship														
Yes	577	74.7	249	63.4	374	73.2	128	68.4	138	91.4	31	46.3	671	73.3
No	159	20.6	82	20.9	122	23.9	52	27.8	12	7.9	19	28.4	205	22.4
Missing	36	4.7	62	15.8	15	2.9	7	3.7	1	0.7	17	25.4	40	4.4
Work rate^a^														
Part-time	—	—	—	—	61	11.9	36	19.3	33	21.9	5	7.5	135	14.7
Full time	—	—	—	—	445	87.1	147	78.6	118	78.1	49	73.1	759	82.9
Missing	—	—	—	—	5	1.0	4	2.1	0	0.0	13	19.4	22	2.4

^a^ Question added in the 2022 questionnaire.

### Variations in Health Problems, Accidents, and Working Conditions

Table 1; Figure 3 present the sample characteristics and prevalence of health problems, and driving accidents in 2010, 2018, and 2022 among BDs affiliated with union 1. The proportion of female bus drivers significantly increased from 4.7% in 2010 to 13.7% in 2022 (OR = 3.77 (2.36, 6.04), *p-*value > 0.001). The average age was 46.4 (±9.0) years in 2010, 45.7 (±13.0) in 2010, and 47.4 (±10.1) in 2022. Shoulder or neck muscle pain was the most prevalent health issue for the three-time points (51.0% in 2010, 51.4% in 2018, and 59.4% in 2022). Table 2 summarizes the changes in health problems and accidents between 2010 and 2022. Headaches, anxiety, and hypersudation decreased during the last decade. However, shoulder or neck muscle pain, sleep disorders, sick leaves, and driving accidents (5.3%–13.4%) increased between 2010 and 2022. The tediousness score of ten out of the twelve analyzed working conditions increased between 2010 and 2022 (Table 3). It corresponds to working days over 10 h, cyclists’ behavior, long periods without access to toilets, driving time over 4 h, the aggressiveness of other road users, difficult traffic conditions, aggressive customers, delayed schedule, traffic disruption, and night work.

**TABLE 2:** Changes in health problems and accidents over 12 years (2010, 2018, and 2022 surveys) in bus drivers affiliated with union 1 (Stratégie Energétique et Santé, Switzerland, 2022).

	2010–2018			2010–2022			2018–2022			Selection for modeling^a^
	OR	95% CI	*p*-value	OR	95% CI	*p*-value	OR	95% CI	*p*-value	
Health problems										
Abnormal fatigue	0.79	0.59, 1.04	0.092	0.99	0.77, 1.27	0.940	1.26	0.94, 1.69	0.123	
Shoulder or neck muscle pain	0.83	0.62, 1.10	0.187	1.14	0.89, 1.47	0.300	1.38	1.03, 1.87	0.034	X
Upper limp muscle pain	0.72	0.50, 1.02	0.067	0.84	0.61, 1.14	0.256	1.16	0.80, 1.69	0.426	
Lower limp muscle pain	0.75	0.52, 1.07	0.110	0.98	0.72, 1.33	0.890	1.31	0.91, 1.89	0.153	
Back pain	0.84	0.63, 1.11	0.220	0.97	0.75, 1.24	0.802	1.15	0.86, 1.55	0.340	
Headache	0.58	0.42, 0.79	0.001	0.67	0.51, 0.88	0.004	1.15	0.82, 1.61	0.404	
Stomach pain	0.87	0.60, 1.27	0.475	0.84	0.60, 1.17	0.303	0.96	0.64, 1.43	0.846	
Stress	0.83	0.62, 1.10	0.187	0.86	0.67, 1.11	0.258	1.05	0.78, 1.41	0.772	
Anxiety	0.66	0.46, 0.96	0.031	0.80	0.58, 1.12	0.195	1.21	0.82, 1.80	0.342	
Irritability	0.90	0.67, 1.20	0.465	1.00	0.77, 1.30	0.985	1.12	0.82, 1.52	0.476	
Sleep problems	1.38	1.04, 1.84	0.026	1.39	1.08, 1.79	0.012	1.00	0.75, 1.35	0.983	X
Appetite or digestion problems	0.89	0.64, 1.24	0.500	1.06	0.80, 1.42	0.674	1.19	0.84, 1.68	0.319	
Hypersudation	0.50	0.30, 0.84	0.008	0.44	0.28, 0.71	0.001	0.88	0.49, 1.59	0.677	
Accident, sick leave, fitness to drive										
Accident	2.67	1.61, 4.45	<0.001	2.36	1.47, 3.78	<0.001	0.88	0.56, 1.40	0.594	X
Sick leave	1.42	1.06, 1.89	0.018	2.10	1.62, 2.71	<0.001	1.48	1.10, 2.00	0.010	X
Unfit to drive	1.22	0.90, 1.64	0.202	0.94	0.72, 1.24	0.670	0.77	0.56, 1.06	0.114	

OR, odd ratio; CI, confidence interval.

This table shows the results of multivariate logistic regression models adjusted for sex, age, seniority, and working region for each outcome. The predictor of interest is the year, which is used as a factor variable. The 2010–2018 and 2010–2022 models use the 2010 year as a reference and the 2018–2022 model uses the 2018 years as a reference.

^a^ Health problems and accidents having either increased over the past 12 years or the past 4 years.

**TABLE 3:** Changes in working conditions between 2010 and 2022 (2010, 2018, and 2022 surveys) according to bus drivers affiliated with union 1 (Stratégie Energétique et Santé, Switzerland, 2022).

	2010–2018			2010–2022			2018–2022		
	OR	95% CI	*p*-value	OR	95% CI	*p*-value	OR	95% CI	*p*-value
Working day >10 h	1.33	1.18, 1.49	<0.001	1.20	1.07, 1.33	0.001	1.10	0.97, 1.24	0.154
Cyclist behavior	1.15	1.01, 1.32	0.041	0.98	0.87, 1.11	0.796	1.17	1.01, 1.35	0.032
Long period without access to toilets	1.30	1.14, 1.49	<0.001	1.42	1.26, 1.59	<0.001	0.92	0.8, 1.05	0.221
Driving time >4 h	1.36	1.19, 1.56	<0.001	1.22	1.08, 1.38	0.001	1.10	0.95, 1.27	0.198
Aggressiveness of other road users	1.34	1.16, 1.54	<0.001	1.19	1.05, 1.35	0.007	1.12	0.96, 1.29	0.148
Difficult traffic conditions	1.57	1.38, 1.8	<0.001	1.39	1.23, 1.56	<0.001	1.12	0.97, 1.29	0.125
Aggressive customers	1.43	1.24, 1.65	<0.001	1.39	1.22, 1.58	<0.001	1.03	0.88, 1.2	0.740
Delayed schedule	1.72	1.49, 1.99	<0.001	1.58	1.39, 1.8	<0.001	1.08	0.93, 1.26	0.330
Traffic disruption	1.74	1.52, 2.01	<0.001	1.47	1.3, 1.66	<0.001	1.18	1.02, 1.37	0.028
Night work	1.17	0.99, 1.38	0.066	1.18	1.02, 1.37	0.027	0.99	0.83, 1.18	0.923
Evening work	1.16	1, 1.34	0.055	1.11	0.98, 1.27	0.106	1.04	0.89, 1.22	0.606
Sunday work	1.08	0.93, 1.25	0.321	1.15	1.01, 1.32	0.036	0.93	0.79, 1.09	0.361

OR, odd ratio, calculated using the increase in 1 point in score of the variable. CI, confidence interval.

This table shows the results of multivariate regression models adjusted for sex, age, seniority, and working region for each outcome. The predictor of interest is the year, which is used as a factor. The 2010-2018 and 2010–2022 models use the 2010 years as a reference and the 2018–2022 model uses the 2018 years as a reference.

### Possible Explanatory Factors of Health Problems and Accidents

The tables summarizing the results of the univariate and multivariate regression models for the shoulder or neck muscle pain models, sleep disorders models, sick leave models, and accident models are presented in Supplementary Tables S1–S4, respectively.

#### Geographical Region

Sleep disorders, sick leaves, and accidents were significantly associated with the geographic region of BDs’ residence. Sleep disorders increased in Zurich [OR = 3.19 (1.31, 7.72)] compared to Espace Mitteland. In addition, sick leaves increased in Lake Geneva Region [OR = 1.63 (1.01, 2.61)]. Accidents were less frequently reported in Lake Geneva Region [OR = 0.33 (0.15, 0.74)] compared to Espace Mitteland.

#### Shoulder or Neck Muscle Pain

Shoulder or neck muscle pain increased with the presence of upper limb muscular pain [OR = 5.58 (3.13, 9.95)], headaches [OR = 1.89 (1.27, 2.81)], and with the driving period over 4 h [OR = 1.43 (1.12, 1.81)].

#### Sleep Disorders

Sleep disorders and sick leaves increased with the presence of anxiety [OR = 1.87 (1.11, 3.16, and OR = 1.65 (1.00, 2.73), respectively]. The prevalence of self-reported sleep disorders and driving accidents was associated with sick leaves [OR = 1.64 (1.11, 2.40), and OR = 2.16 (1.26, 3.72), respectively]. Sleep disorders were less prevalent among BDs affiliated with union 3 *versus* union 1 [OR = 0.47 (0.26, 0.86)] but more prevalent among BDs with superior school level *versus* mandatory school level [OR = 1.72 (1.02, 2.88)]. Their prevalence of sleep disorders increased in the presence of stomach pain [OR = 2.02 (1.14, 3.58)], abnormal fatigue [OR = 2.41 (1.63, 3.55)], and back pain [OR = 1.49 (1.02, 2.17)].

#### Driving Accidents

Driving accidents were three-fold more frequent among BDs who made an apprenticeship of another occupation before becoming BD than BDs who did not [OR = 3.00 (1.42, 6.36)]. BDs driving regional lines, or a mix of regional and urban lines reported fewer accidents [OR = 0.37 (0.16, 0.88), and OR = 0.53 (0.28, 0.99), respectively].

#### Sick Leaves

Sick leave prevalence increased with seniority [1.03 per additional year (1.00, 1.05)], the presence of sleep disorders [1.71 (1.17, 2.50)], and the accident reporting in the last 12 months [OR = 2.16 (1.01, 1.62)].

#### SARS-CoV-2 Potential Consequences

In most cases, when adding the SARS-CoV-2 consequences on working conditions in the multivariate models, the associations found in the first multivariate model remained and some new ones appeared (Supplementary Tables S4–S7). The prevalence of sleep disorders, sick leaves, and accidents increased with SARS-CoV-2 crisis consequences on physical or mental health [OR = 1.83 (1.11, 3.00), OR = 1.82 (1.14, 2.91), and OR = 3.05 (1.51, 6.14), respectively]. In addition, sleep disorders increased with seniority [1.03 per additional year (1.01, 1.06)]. Shoulder or neck muscle pain was more prevalent among female BDs *versus* male BDs [OR = 1.81 (1.04, 3.17)].

## Discussion

### Main Findings

Musculoskeletal disorders were the most prevalent subjective health issue reported in this study. This issue was reported in many studies in many countries [2, 14, 15, 18, 20, 29, 32, 33] and their causes are well known: vibrations, awkward seating, and long period seating [2, 15, 18–21]. The prevalence of nine out of the sixteen health issues and accident studied has not changed across the three time-points despite important changes in BDs’ working conditions (Table 3). Only three health issues have a decreased prevalence. More frequent use of vehicles equipped with air conditioning could be an explanation for a decrease in hypersudation prevalence.

### Result Interpretation

Our assumption in logistic regression modeling was that more BDs consider their work conditions as tedious more frequently the associated health condition would be reported. We did not have information on the frequency of tedious work situations. However, some of them may be repetitive, such as passengers’ aggressiveness which can occur several times a week or even daily [49].

#### Musculoskelettal Disorders

In the explanatory model of shoulder or neck muscle pain, the associated working condition was the driving shift longer than 4 h, which seems perfectly plausible, considering all the other factors as independent. While a working day longer than 10 h was also associated with this health outcome, with a similar effect size as >4-h driving shift in the univariate analysis, in the adjusted models, this association disappeared because of collinearity with the long driving shifts. The same is true for the effect size of anxiety and stress, which are similar and statistically significant in the univariate analysis, but change in the fully adjusted models. Besides these working conditions, the reporting of the upper limb muscular pain and headache also explained the presence of shoulder or neck muscle pain, with quite high ORs (Supplementary Table S1). Furthermore, a two-fold increased risk of shoulder or neck muscle pain associated with female sex is another important finding, considering the growing feminization of the bus driver profession. For a long time, all bus drivers were men, and the vehicles were designed according to their stature. Presently, there are four times as many female BDs as in 2010 and the buses are not adapted to their, usually smaller, stature. This finding should be considered in the design of new vehicles. Finally, an observed association between shoulder or neck muscle pain and passengers’ aggressiveness in the fully adjusted deserves a cautious interpretation. In the univariate model, a statistically non-significant OR of 1.10 was observed. In the adjusted model, it first decreased to 0.79, then to 0.73, which became significant, but more as a statistical artifact than a meaningful finding. While it can be a risk factor for the outcomes considered in this study, when controlled for all the other variables identified in the DAG, it disappears and/or changes direction likely due to an over-adjustment. Considering passengers or other road users’ aggressiveness as protective against shoulder or neck muscle pain would be completely misleading, especially because this effect is observed only when adjusting for SARS-CoV-2 impacts. It is thus likely, that the measures taken against the spread of SARS-CoV-2 had a protective effect on the bus driver who was thus isolated from the unkind passengers and protected from their aggression.

#### Sleep Disorders

An important finding was the association of seniority with an increased prevalence of sleep disorders. We expected the cumulated exposure to various hazards to increase with increasing seniority, which in turn increases the risk of having sleep disorders. Studies have shown an increase in mental health issues, including stress, due to the SARS-CoV-2 pandemic [25, 50]. As known, stress could deteriorate sleep quality [51]. Sleep disorders were three times more frequent in Zurich than in the Espace Mitteland. This could be explained by different working conditions or different sensitivities to sleep deprivation. To be noted, union 1 did a prevention campaign focused on sleep disorders at one of the companies corresponding to one-third of the participants living in Zurich. This might explain the association of a higher prevalence of sleep disorders with affiliation with union 3, as BDs part of union 3 might be more indulgent or less sensitive to this problem.

#### Sick Leaves

In the explanatory model of sick leaves, the associated working conditions were the working day over 10 h and the driving shift longer than 4 h, which seems perfectly plausible as this corresponds to longer exposure to hazards during a day. The association of anxiety and stress, which were statistically significant and led to an excess risk of sick leaves in univariate analysis, changed in the fully adjusted models. Indeed, stress usually negatively impacts health, increasing the risk of sleep disorders or stomach pain [51, 52]. However, in our study, anxiety, which is highly correlated with stress, explains better the sick leaves, anxious BDs tend to have more sick leaves. The cumulated exposure to various hazards increases with increasing seniority, which in turn increases the risk of having a disease and being on sick leave. The association of sick leaves with SARS-CoV-2 impact on physical or mental health was expected as bus drivers were at higher risk to contract the disease [24, 26] and the pandemic was linked to an increase in mental disorders in essential workers [25, 49, 50]. Among unions, Lake Geneva Region was known to have a problem with high absenteeism. Unions explained that due to the shortage of BDs [7, 11] and to maintain the service, bus companies had to make the present staff work, even if it meant eliminating days of rest. This results in an overload of work that could lead to employees taking a couple of sick leave days because they were no longer able to work.

#### Driving Accidents

Urban service was associated with excess risk of driving accidents. The working environment is different for urban and regional routes. Urban traffic is often considered more stressful with heavier traffic, more bicycles, more pedestrians, more disruptions on the road, and a tighter schedule. All these elements can be stressors and cause traffic accidents. In contrast, regional service generally has less traffic, fewer disruptions, and a looser schedule with more break time at the terminus. In addition, according to many drivers and unions, passenger behavior is also different between the two types of services. In the cities, people are usually more stressed and aggressive than outside the cities [53].

In both univariate and multivariate logistic regression models (Supplementary Table S4) the increased prevalence of driving accidents was associated with apprenticeship. To understand this association, it is important to understand the Swiss educational system, composed of three levels: compulsory (mandatory school), upper secondary (apprenticeship or general education), and tertiary (universities or college). During mandatory school, depending on their school grades, students are divided into sectors, either extended requirements or basic-requirement. After mandatory school, students from the basic-requirement sector do an apprenticeship, while the others can either continue their study or do an apprenticeship. Overall, in Switzerland, two-thirds of young people choose to do an apprenticeship [54, 55]. The BD profession is not an apprenticeship in Switzerland, but a training acquired in employment in the bus company for 6 months to 1 year. To start the BD training, it is mandatory to have an apprenticeship or at least 2 years of professional experience. All Swiss BDs had previous work experience, which might have no link with driving such as cook or jurist. In addition, apprenticeships are more frequent for occupations that are more physically demanding and exposed than occupations accessible only through higher education.

Contrary to before, in the univariate models, stress had a stronger effect than anxiety, and after adjustment, stress kept an excess risk (OR>1) while anxiety lost its effect when adding stress to the model. This seems plausible as stress is a known cause of driving accidents as it might lead to loss of focus and induce fatigue [56]. Fatigue is another known cause of driving accidents [31], we found that fatigue led to an excess risk (OR>1) of accidents in our univariate model. Moreover, the impacts of the SARS-CoV-2 pandemic on health and sick leave were partly related to mental health and fatigue, both of which were risk factors for accidents [31, 56].

### Results Generalization and Risk of Bias

As both the exposures and the outcome were self-reported, some participants could have over or under-reported their exposure and health problems. This could lead to an information bias, which is present in all studies of this kind [57]. Without additional measurement data on these variables, it is impossible to predict the amount and direction of a potential misclassification bias. As we did not ask about the occupation learned in apprenticeship and the frequency of tedious work conditions, the study does not allow any causal understanding of the health outcomes and is a hypotheses-generating study. A selection bias might be present as there could be differences between the participants and the BDs who did not participate. Having only unionized drivers, which corresponds to 40.3% of the Swiss BDs, they could not represent the whole Swiss BD population, our target population. Noteworthy, the unionization depends on the company, varying from 5% to 80%. The under-representation of German-speaking region and the over-representation of the French and Italian-speaking regions is typical situation in similar surveys conducted in Switzerland [58], as we discussed elsewhere [45]. As the distribution of our sample does not perfectly reflect the target population of Swiss BDs and due to the design and availability of data, the results of this study should be considered as preliminary and further confirmed in methodologically stronger studies.

### Conclusion

This study showed that many Swiss BDs report multiple health problems simultaneously and this trend has worsened between 2010 and 2022. The most frequently perceived health problems are musculoskeletal diseases including shoulder or neck muscle pain or back pain. The second most frequent complaint is abnormal fatigue reported by more than half of the BDs. Shoulder or neck muscle pain, sleep disorders, sick leaves, and accidents have increased since 2010 or 2018. The increased health issues were associated with working conditions, which also worsened in the 12 past years and the presence of co-morbidity. The SARS-CoV-2 pandemic had additional negative consequences on BD’s health outcomes. The study was based on self-reported data by unionized BDs. Therefore, the results deserve a cautious interpretation and generalization. Nevertheless, they suggest a need for cohort studies to confirm these findings and to set up interventions targeting the most tedious and harmful work conditions, such as long driving/working hours.
