# Supplementary material for: Healthy Bus Drivers, Sustainable Public Transport: A Three-Time Repeated Cross-Sectional Study in Switzerland
Source: Int J Public Health. 2023 Jun 21;68:1605925. doi: 10.3389/ijph.2023.1605925 (PMC10319994; doi:10.3389/ijph.2023.1605925)
Supplement: Supplementary file 1 [file DataSheet1.pdf]

Supplementary File 1: Questionnaire 2022

Langue/ Sprache/ Lingua : ☐ French ☐ Deutsch ☐ Italiano

HEALTH AND WORK

The unions SEV, syndicom, and SSP, together with researchers from Unisanté, are launching a survey among public transport drivers about occupational safety and health protection.
This survey takes the questions from the previous SEV surveys of 2010 and 2018 to make a global analysis of the evolution of the working conditions and your health status. As a result, it includes a question about your career as a driver and three questions to get your opinion about a more in-depth study project, in partnership with Unisanté researchers.
The results will be used to strengthen our union action in the field of health prevention and to put forward our demands during future conventional negotiations.
This survey is **completely anonymous**. Regardless of your answers, no respondent can be identified, even if several questions are crossed. The results will be presented and discussed at union meetings.

Health

Do you experience problems with the following at least once a month?
Please check the problem(s) you are experiencing

- ☐ Abnormal fatigue
- ☐ Muscle pain in the shoulders or neck
- ☐ Muscle pain in the upper limbs
- ☐ Muscle pain in the lower limbs
- ☐ Back pain
- ☐ Headaches
- ☐ Stomach pain
- ☐ Stress
- ☐ Anxiety
- ☐ Irritability
- ☐ Sleep disorders
- ☐ Appetite or digestion problems
- ☐ Hypersudation (heavy sweating)

Working conditions tediousness

Please check the problem(s) you are experiencing

Very tedious	Tedious	Not very tedious	Not tedious	
☐	☐	☐	☐	Night work (from 10 p.m.)
☐	☐	☐	☐	Evening work (from 6 p.m.)
☐	☐	☐	☐	Sunday’s work
☐	☐	☐	☐	Traffic conditions
☐	☐	☐	☐	Driving time of more than four hours
☐	☐	☐	☐	Range of days longer than 10 hours
☐	☐	☐	☐	Delayed schedule
☐	☐	☐	☐	The aggressiveness of users (clients)
☐	☐	☐	☐	Aggression from other road users
☐	☐	☐	☐	Traffic disruption (accident/parking for delivery)
☐	☐	☐	☐	Cyclist behavior
☐	☐	☐	☐	Long periods without access to the toilet

Ergonomics

How important is it to you to:

	1-Not important	2	3	4	5- Very important
The quality of the seat	☐	☐	☐	☐	☐
Adjustment and arrangement of the driving instruments (steering wheel, etc.)	☐	☐	☐	☐	☐
Closing the cabin	☐	☐	☐	☐	☐
Ventilation and air conditioning	☐	☐	☐	☐	☐
Closing the front door	☐	☐	☐	☐	☐
Airflow	☐	☐	☐	☐	☐
Noise	☐	☐	☐	☐	☐

Work environment

Can you tell us what is going well at work? (Maximum three criteria)

- ☐ Solidarity between workers
- ☐ Correct superiors
- ☐ Quality of work
- ☐ Job satisfaction
- ☐ Freedom, independence
- ☐ Employment secured
- ☐ Salary
- ☐ Schedules
- ☐ Other: Please specify: ____

Accidents and illnesses

Have you had to miss work for health reasons in the past year?

☐ Yes ☐ No

Did you suffer a lost time injury at work in 2021?

☐ Yes ☐ No

Did you have an illness that you feel is related to your work in 2021?

☐ Yes ☐ No

Do you always drive while in full control?

☐ Yes ☐ No

If not, why not?

____

You personally

This information is **essential** for understanding the results of the survey. It **will not be processed individually** but will be grouped with all the responses obtained, **to make analyses by subgroups** according to the criteria listed below.

What is your gender? ☐ Male ☐ Female

How old are you? _____________

What is your level of education? □ Mandatory school □ Secondary school □ University

Have you done an apprenticeship? □ Yes □ No

In which canton do you reside:

□Appenzell Rh.-Ext.	□Appenzell Rh.-Int.	□Aargau	□Basel-Landschaft
□Basel-City	□Bern	□Fribourg	□Geneva
□Glarus	□Grisons	□Jura	□Lucerne
□Neuchâtel	□Nidwalden	□Obwalden	□St. Gallen
□Schaffhausen	□Schwyz	□Solothurn	□Ticino
□Thurgau	□Uri	□Valais	□Vaud
□Zug	□Zurich		

Your professional career

The purpose of this question is to **retrace your driving history in** more detail.
What company do you currently work for? ____________________________ (drop-down list of choices)

How long have you been working there? *Approximate date (DD-MM-YYYY)* _______________________________

Do you work full-time (100%)? □ Yes □ No

If not, what rate do you work? *(Indicate the rate as a percentage. Example 40%)* ________________

What type of line do you operate? □ Urban □ Regional □ Mixed

Did you work for another public transportation company? □ Yes □ No

If yes: the same similar set of questions

If not: nothing is displayed, section completed

Your opinion on the Covid situation

From your perspective, did your employer respond quickly enough at the onset of the pandemic (i.e., early 2020) to protect you from the coronavirus in your workplace?

☐ Yes ☐ No

In your opinion, are you sufficiently protected against the coronavirus at your workplace today?

☐ Yes ☐ No

From your point of view, are the concepts of protection against coronavirus well implemented in your workplace?

☐ Yes ☐ No

Did you have to work a lot of extra hours because of the coronavirus (e.g., filling in for colleagues)?

☐ Yes ☐ No

Have you ever had to take over shifts from your colleagues on short notice because of the coronavirus?

☐ Yes ☐ No

Has your workload increased overall because of the coronavirus?

☐ Yes ☐ No

Has the extra work resulting from the pandemic led to a shortening of your rest periods?

☐ Yes ☐ No

Has the increased workload had an impact on your psychological or physical state?

☐ Yes ☐ No

How would you rate your employer's handling of the coronavirus after two years of the pandemic?

☐ 1 ☐ 2 ☐ 3 ☐ 4 ☐ 5 ☐ 6

The unions SEV, syndicom, and SSP and the Unisanté team would like to thank you for your commitment and cooperation!

Supplementary File 2: Direct Acyclic Graphs (DAGs)

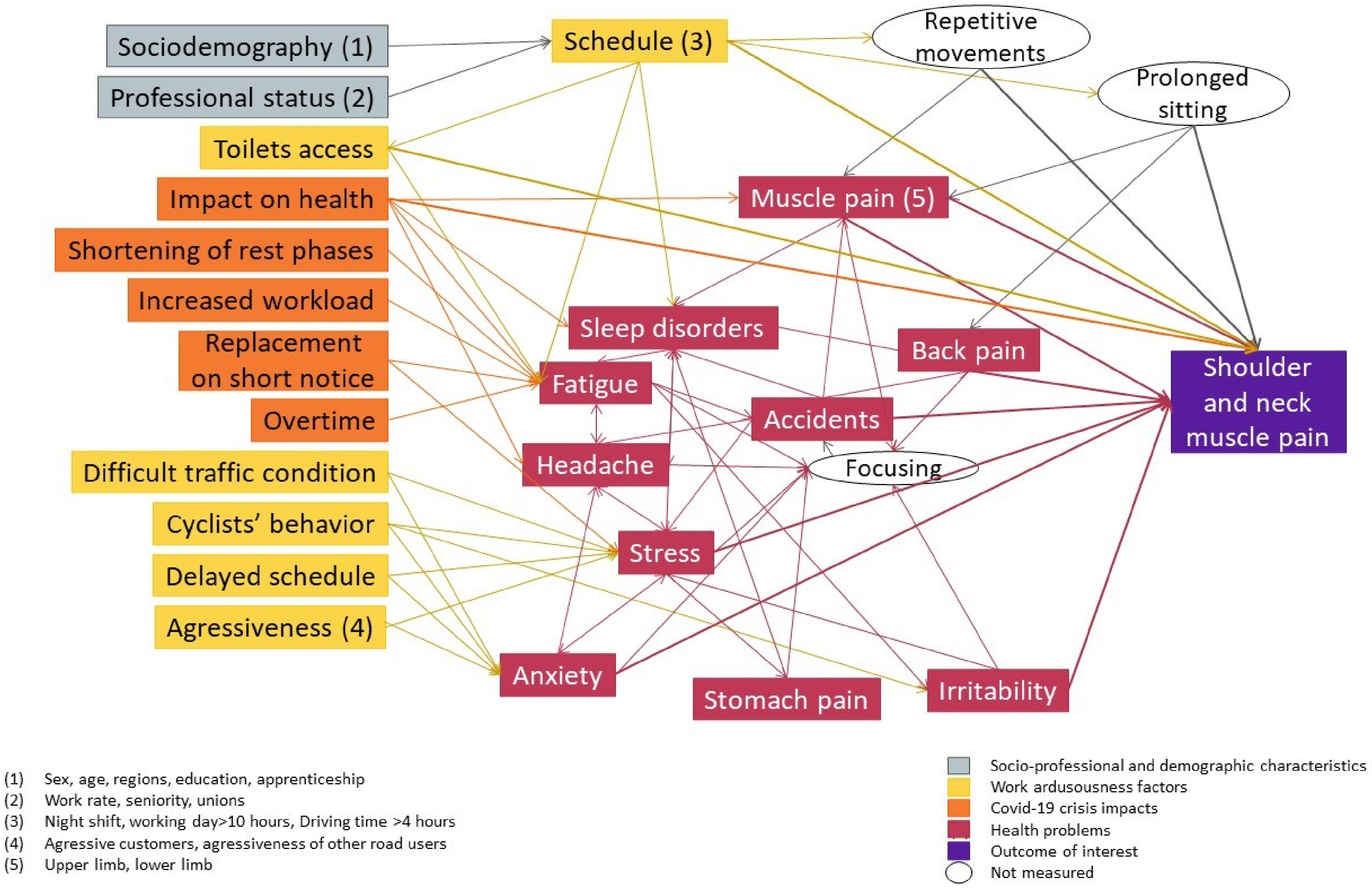

Figure S. 1. DAG for shoulder and neck muscle pain. This includes factors that can lead to poor posture over a prolonged period, repetitive movements, and factors that can cause upper body tension and tightness such as stress. We also include accidents and the impact of the SARS-CoV-2 crisis that can cause muscle pain. (Stratégie Energétique et Santé, Switzerland, 2022)

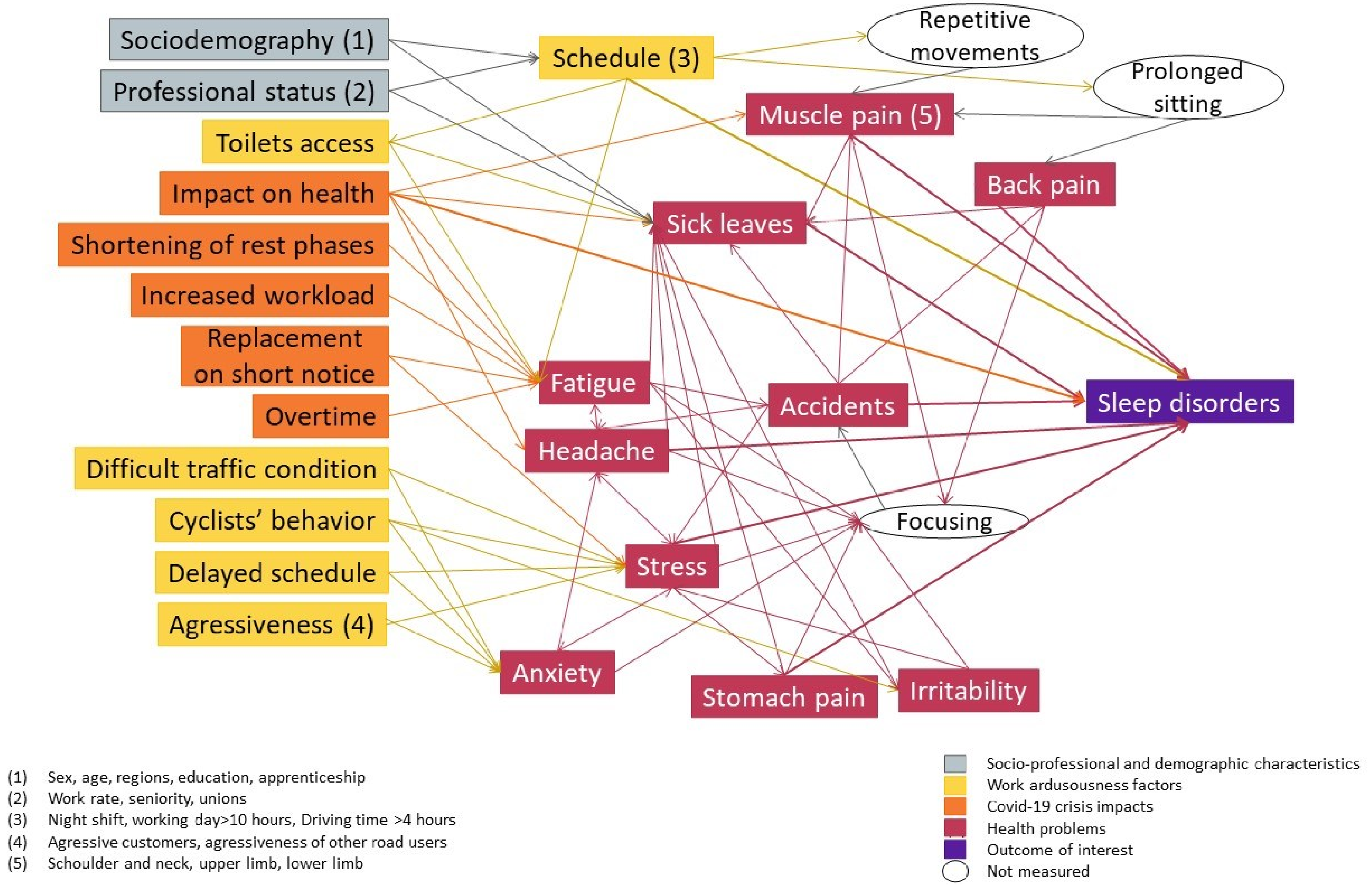

Figure S. 2. DAG for sleep disorders. This includes factors that can cause pain that prevents you from falling asleep, schedule changes that can disrupt the circadian cycle and stress factors that can also influence sleep disorders. (Stratégie Energétique et Santé, Switzerland, 2022)

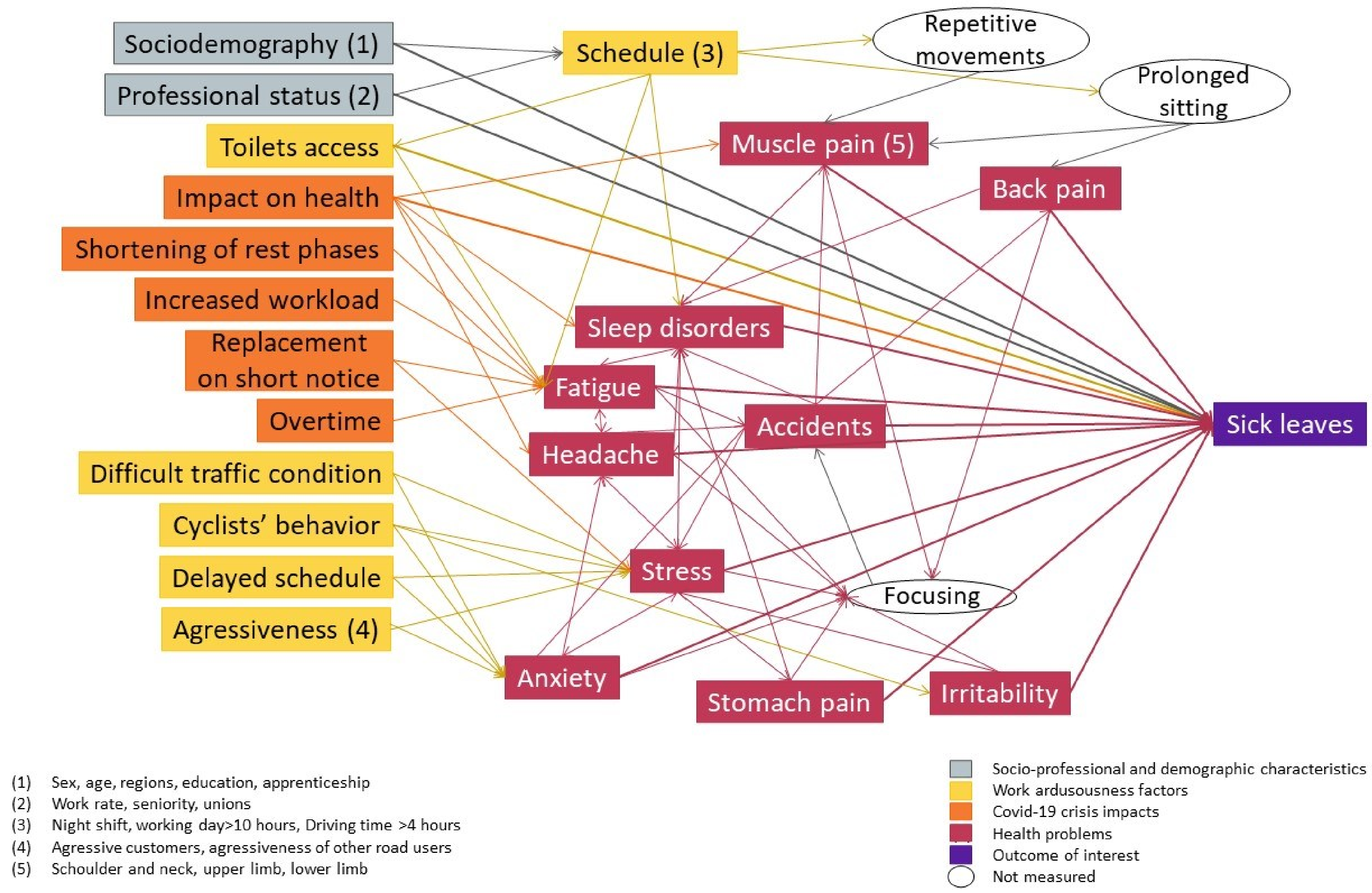

Figure S. 1. DAG for sick leaves. This includes factors that may be related to illnesses that would result in sick leave for physical health reasons (e.g., musculoskeletal disorders) or mental health reasons (e.g., burnout). (Stratégie Energétique et Santé, Switzerland, 2022)

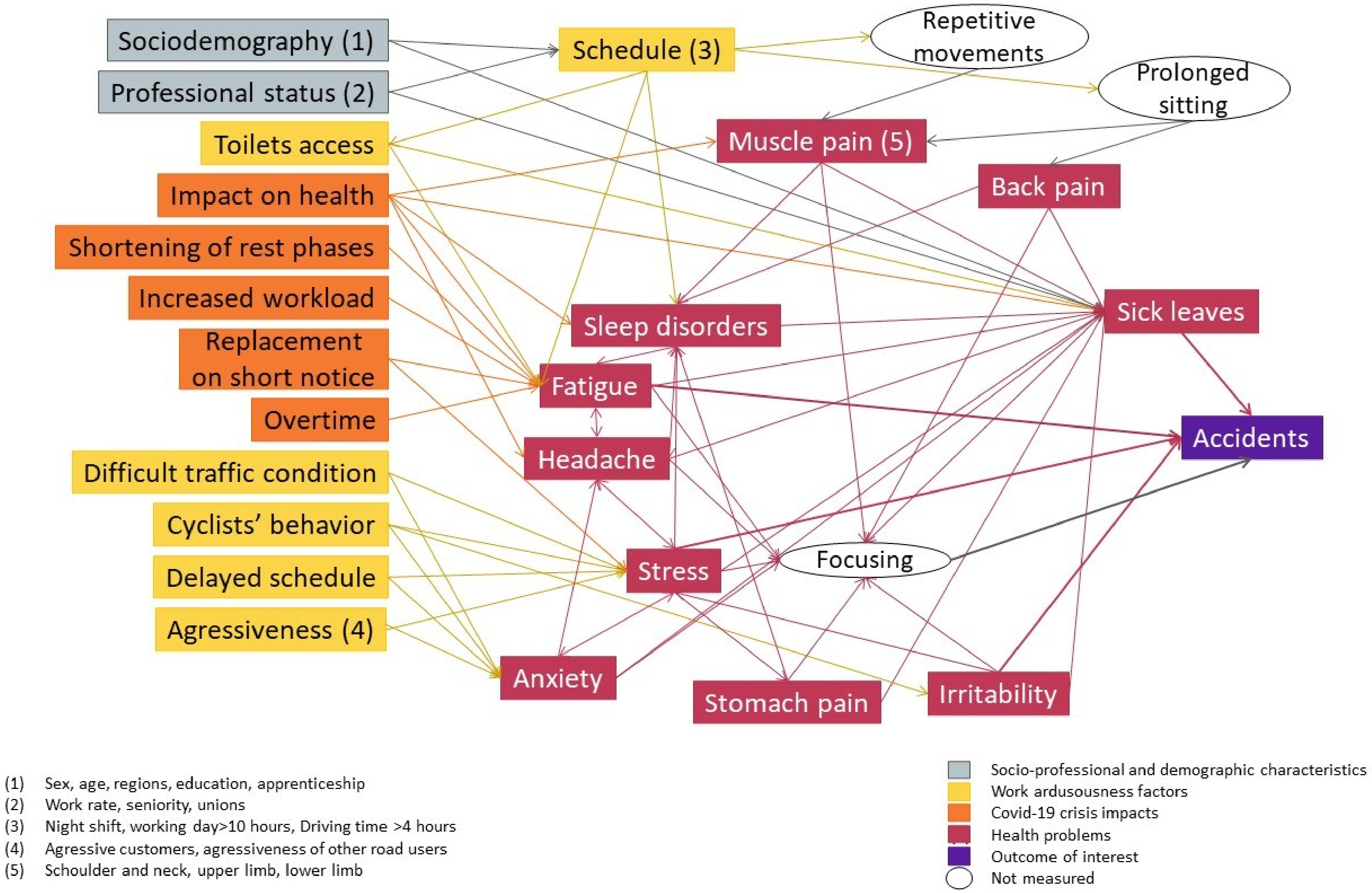

Figure S. 4. DAG for accidents. This includes factors that may be related to impaired concentration (e.g., pain) or attention, the risk of falling asleep at the wheel, and any factors that may make a driver unfit to drive (Stratégie Energétique et Santé, Switzerland, 2022).

Supplementary File 3: Distribution of unions and participants per region

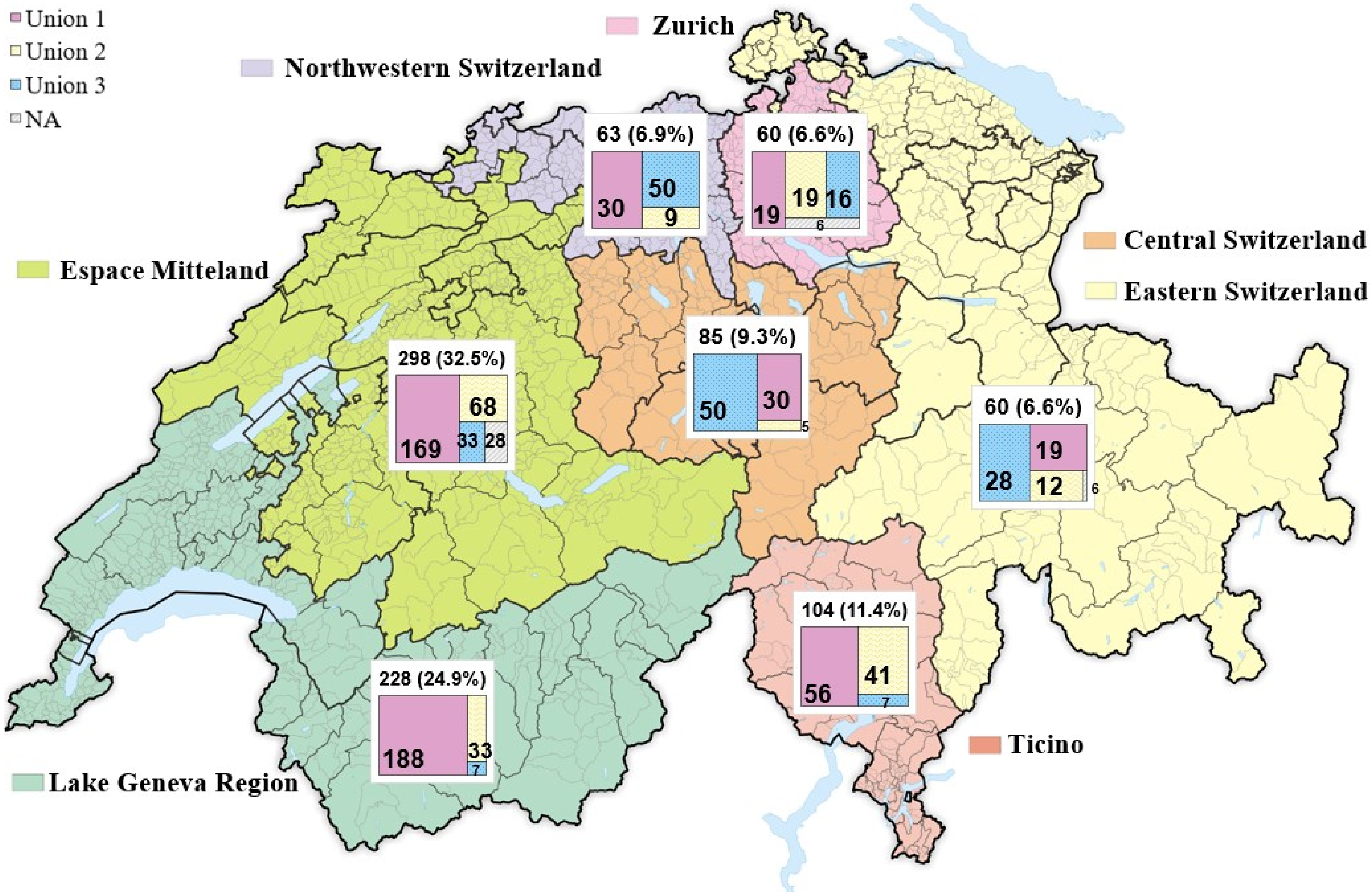

Figure S.5. Distribution of unions and participants (N) per region (2022 sample) (Stratégie Energétique et Santé, Switzerland, 2022)

Supplementary File 4 : Results of univariate and multivariate logistic regression models

Table. S. 1. Explanatory factors of shoulder or neck muscle pain (2022 survey among unionized bus drivers, Switzerland) (Stratégie Energétique et Santé, Switzerland, 2022)

	Model 1 *			Model 2 **			Model 3 ***		
	OR	95% C.I.	p-value	OR	95% C.I.	p-value	OR	95% C.I.	p-value
Female sex	1.77	1.19, 2.64	0.005	1.66	0.98, 2.81	0.061	1.79	1.03, 3.14	0.040
Age (per year)	1.00	0.99, 1.01	0.884	1.01	0.99, 1.03	0.448	1.00	0.98, 1.02	0.786
Seniority (per year)	1.00	0.98, 1.01	0.789	0.99	0.97, 1.01	0.445	0.99	0.97, 1.01	0.519
Espace Mitteland	Ref.	Ref.	Ref.	Ref.	Ref.	Ref.	Ref.	Ref.	Ref.
Northwestern Switzerland	0.71	0.41, 1.23		0.76	0.37, 1.55		0.75	0.36, 1.56	
Eastern Switzerland	1.10	0.63, 1.93		1.79	0.84, 3.80		1.81	0.83, 3.97	
Lake Geneva Region	1.40	0.98, 1.99	0.002	1.01	0.63, 1.62	0.370	0.89	0.54, 1.46	0.385
Ticino	1.16	0.74, 1.82		1.03	0.54, 1.94		0.92	0.47, 1.81	
Central Switzerland	0.52	0.32, 0.85		0.68	0.35, 1.33		0.73	0.36, 1.47	
Zurich	1.83	1.01, 3.33		1.44	0.65, 3.19		1.55	0.68, 3.57	
Union 1	Ref.	Ref.	Ref.	Ref.	Ref.	Ref.	Ref.	Ref.	Ref.
Union 2	0.82	0.58, 1.15	0.241	0.82	0.51, 1.32	0.419	0.89	0.54, 1.46	0.643
Union 3	0.79	0.55, 1.14	0.201	0.98	0.57, 1.70	0.949	0.99	0.56, 1.74	0.973
Mandatory School	Ref.	Ref.	Ref.	Ref.	Ref.	Ref.	Ref.	Ref.	Ref.
Secondary School	1.14	0.84, 1.54	0.405	0.91	0.61, 1.35	0.630	0.97	0.64, 1.46	0.868
Superior School	1.13	0.81, 1.6	0.471	0.95	0.59, 1.53	0.830	0.97	0.59, 1.59	0.894
Apprenticeship	0.82	0.6, 1.13	0.225	1.01	0.66, 1.54	0.967	1.01	0.65, 1.57	0.973
Full-time	Ref.	Ref.	Ref.	Ref.	Ref.	Ref.	Ref.	Ref.	Ref.
Part-time	0.70	0.48, 1	0.052	0.71	0.43, 1.18	0.190	0.71	0.42, 1.21	0.208
Stress	1.84	1.41, 2.41	<0.001	1.07	0.72, 1.57	0.749	1.16	0.77, 1.74	0.469
Anxiety	2.27	1.55, 3.33	<0.001	1.54	0.91, 2.6	0.107	1.68	0.97, 2.90	0.063
Irritability	1.55	1.17, 2.05	0.002	1.15	0.78, 1.71	0.475	1.14	0.76, 1.71	0.515
Upper limp muscular pain	9.73	5.85, 16.16	<0.001	5.54	3.11, 9.89	<0.001	5.14	2.85, 9.26	<0.001
Fatigue	1.36	1.04, 1.76	0.022	0.81	0.55, 1.18	0.263	0.88	0.59, 1.3	0.510
Sleep disorders	1.93	1.48, 2.52	<0.001	1.27	0.87, 1.86	0.221	1.26	0.85, 1.87	0.255
Headache	2.47	1.83, 3.33	<0.001	1.93	1.30, 2.87	0.001	2.12	1.41, 3.18	<0.001
Stomach pain	1.93	1.29, 2.88	0.001	1.17	0.67, 2.04	0.576	0.99	0.56, 1.76	0.980
Accident	1.80	1.2, 2.7	0.005	1.20	0.7, 2.05	0.503	1.37	0.78, 2.41	0.273
Working day > 10 hours	1.46	1.24, 1.72	<0.001	1.01	0.78, 1.31	0.924	1.03	0.79, 1.35	0.808
Driving time > 4 hours	1.61	1.39, 1.86	<0.001	1.43	1.13, 1.82	0.003	1.50	1.17, 1.92	0.001
Aggressive customers	1.11	0.96, 1.28	0.152	0.80	0.61, 1.05	0.113	0.74	0.56, 0.99	0.041
Aggressiveness of other road users	1.23	1.06, 1.43	0.007	1.32	0.96, 1.82	0.090	1.38	0.99, 1.92	0.059
Cyclist behavior	1.18	1.01, 1.38	0.043	0.94	0.73, 1.21	0.616	0.95	0.73, 1.23	0.692
Long periods without access to toilets	1.30	1.12, 1.5	0.001	1.07	0.85, 1.33	0.578	1.05	0.83, 1.33	0.673
Delayed schedule	1.17	1.00, 1.36	0.046	0.92	0.74, 1.15	0.489	0.96	0.77, 1.21	0.757
Complicated condition	1.29	1.11, 1.49	0.001	1.04	0.8, 1.34	0.770	0.99	0.76, 1.28	0.916
Impact on health	1.67	1.27, 2.2	<0.001	-	-	-	1.06	0.66, 1.70	0.804
Shortening of rest phase	1.37	1.04, 1.81	0.024	-	-	-	1.25	0.76, 2.05	0.383
Overtime	1.09	0.84, 1.43	0.514	-	-	-	1.02	0.65, 1.59	0.928
Increased workload	1.18	0.9, 1.55	0.233	-	-	-	0.65	0.4, 1.06	0.083
Replacement on short notice	0.88	0.67, 1.16	0.380	-	-	-	1.09	0.73, 1.63	0.679

OR: Odd ration, C.I.: confidence interval
* Univariate logistic regression model
** Logistic regression model adjusted for sex, age, driving seniority, region, union, education, and work rate, based on the DAG 1 without SARS-CoV-2 crisis impacts
*** logistic regression model adjusted for sex, age, driving seniority, region, union, education, and work rate, based on the DAG 1 with SARS-CoV-2 crisis impacts

Table. S. 2. Explanatory factors of sleep disorders (2022 survey among unionized bus drivers, Switzerland) (Stratégie Energétique et Santé, Switzerland, 2022)

	**Model 1 ***			**Model 2 ****			**Model 3 *****		
	**OR**	**95% C.I.**	**p-value**	**OR**	**95% C.I.**	**p-value**	**OR**	**95% C.I.**	**p-value**
Female sex	1.05	0.73, 1.53	0.785	1.49	0.87, 2.54	0.147	1.51	0.85, 2.68	0.159
Age (per year)	1.00	0.99, 1.02	0.711	1.01	0.99, 1.03	0.323	1.01	0.98, 1.03	0.617
Seniority (per year)	1.01	1.00, 1.03	0.073	1.02	1.00, 1.05	0.065	1.03	1.01, 1.06	0.014
Espace Mitteland	Ref.	Ref.	Ref.	Ref.	Ref.	Ref.	Ref.	Ref.	Ref.
Northwestern Switzerland	0.88	0.51, 1.53	0.009	1.00	0.46, 2.17	0.007	1.04	0.46, 2.35	0.003
Eastern Switzerland	1.18	0.67, 2.05		1.91	0.85, 4.26		2.14	0.93, 4.91	
Lake Geneva Region	0.97	0.68, 1.37		0.63	0.38, 1.04		0.61	0.36, 1.03	
Ticino	1.54	0.98, 2.42		1.41	0.73, 2.73		1.59	0.80, 3.16	
Central Switzerland	0.55	0.33, 0.91		0.94	0.44, 1.97		0.99	0.45, 2.15	
Zurich	1.76	1.00, 3.10		3.19	1.31, 7.72		3.55	1.41, 8.94	
Union 1	Ref.	Ref.	Ref.	Ref.	Ref.	Ref.	Ref.	Ref.	Ref.
Union 2	1.28	0.91, 1.79	0.153	0.87	0.53, 1.43	0.571	0.84	0.50, 1.41	0.511
Union 3	0.55	0.38, 0.81	0.002	0.47	0.26, 0.86	0.015	0.43	0.23, 0.8	0.008
Mandatory School	Ref.	Ref.	Ref.	Ref.	Ref.	Ref.	Ref.	Ref.	Ref.
Secondary School	1.12	0.83, 1.52	0.455	1.26	0.83, 1.93	0.282	1.31	0.85, 2.04	0.225
Superior School	1.34	0.95, 1.88	0.094	1.72	1.02, 2.88	0.041	1.69	0.99, 2.89	0.056
Apprenticeship	0.86	0.63, 1.17	0.333	0.70	0.45, 1.09	0.111	0.65	0.41, 1.02	0.062
Full-time	Ref.	Ref.	Ref.	Ref.	Ref.	Ref.	Ref.	Ref.	Ref.
Part-time	1.05	0.73, 1.52	0.781	1.44	0.84, 2.48	0.188	1.57	0.89, 2.74	0.117
Stress	2.37	1.81, 3.1	<0.001	1.38	0.92, 2.07	0.115	1.23	0.80, 1.87	0.346
Anxiety	3.50	2.4, 5.11	<0.001	1.87	1.11, 3.16	0.018	1.84	1.07, 3.17	0.027
Irritability	2.24	1.69, 2.95	<0.001	1.38	0.93, 2.05	0.112	1.28	0.85, 1.93	0.244
Headache	1.86	1.41, 2.47	<0.001	1.23	0.82, 1.84	0.325	1.20	0.79, 1.81	0.402
Back pain	2.05	1.58, 2.67	<0.001	1.49	1.02, 2.17	0.040	1.52	1.02, 2.25	0.038
Shoulder and neck pain	1.93	1.48, 2.52	<0.001	1.08	0.72, 1.61	0.711	1.07	0.71, 1.62	0.756
Upper limp muscular pain	2.31	1.65, 3.22	<0.001	1.19	0.73, 1.95	0.485	1.11	0.66, 1.85	0.699
Lower limp muscular pain	1.88	1.34, 2.62	<0.001	1.11	0.68, 1.80	0.684	1.12	0.68, 1.85	0.646
Stomach pain	2.90	1.95, 4.31	<0.001	2.02	1.14, 3.58	0.016	1.86	1.03, 3.35	0.039
Fatigue	3.76	2.85, 4.95	<0.001	2.41	1.63, 3.55	<0.001	2.25	1.50, 3.37	<0.001
Accident	1.66	1.13, 2.45	0.009	0.90	0.53, 1.56	0.717	0.70	0.40, 1.24	0.222
Sick leave	2.21	1.69, 2.89	<0.001	1.64	1.11, 2.40	0.012	1.51	1.01, 2.26	0.044
Night shift	1.44	1.27, 1.64	<0.001	1.03	0.86, 1.25	0.719	1.03	0.85, 1.25	0.766
Working day > 10 hours	1.75	1.47, 2.09	<0.001	1.23	0.92, 1.64	0.163	1.16	0.86, 1.57	0.343
Driving time > 4 hours	1.56	1.35, 1.8	<0.001	1.04	0.80, 1.36	0.743	1.07	0.82, 1.40	0.623
Aggressive customers	1.26	1.09, 1.45	0.002	1.27	0.95, 1.69	0.106	1.26	0.93, 1.70	0.129
Aggressiveness of other road users	1.13	0.98, 1.31	0.103	0.66	0.47, 0.92	0.015	0.67	0.47, 0.94	0.022
Cyclist behavior	1.25	1.07, 1.47	0.006	1.29	1.00, 1.68	0.052	1.29	0.98, 1.68	0.065
Long periods without access to toilets	1.31	1.13, 1.53	<0.001	1.09	0.86, 1.38	0.457	1.03	0.81, 1.32	0.818
Delayed schedule	1.24	1.06, 1.44	0.006	1.00	0.79, 1.27	1.000	0.99	0.78, 1.27	0.962
Complicated condition	1.29	1.12, 1.50	0.001	1.00	0.77, 1.31	0.980	1.02	0.78, 1.35	0.879
Impact on health	3.10	2.34, 4.09	<0.001	-	-	-	1.83	1.11, 3.00	0.017
Shortening of rest phase	1.76	1.34, 2.32	<0.001	-	-	-	1.01	0.59, 1.71	0.975
Overtime	1.34	1.02, 1.75	0.033	-	-	-	1.36	0.85, 2.19	0.199
Increased workload	1.47	1.12, 1.93	0.005	-	-	-	0.66	0.38, 1.12	0.120
Replacement on short notice	1.07	0.81, 1.40	0.643	-	-	-	0.64	0.41, 0.98	0.041

OR: Odd ration, C.I.: confidence interval

* Univariate logistic regression model

** Logistic regression model adjusted for sex, age, driving seniority, region, union, education, and work rate, based on the DAG 2 without SARS-CoV-2 crisis impacts

*** logistic regression model adjusted for sex, age, driving seniority, region, union, education, and work rate, based on the DAG 2 with SARS-CoV-2 crisis impacts

Table. S. 3. Explanatory factors of sick leaves (2022 survey among unionized bus drivers, Switzerland) (Stratégie Energétique et Santé, Switzerland, 2022)

	Model 1 *			Model 2 **			Model 3 ***		
	OR	95% C.I.	p-value	OR	95% C.I.	p-value	OR	95% C.I.	p-value
Female sex	1.18	0.81, 1.72	0.390	1.25	0.75, 2.09	0.392	1.42	0.83, 2.43	0.203
Age (per year)	0.99	0.98, 1.00	0.195	0.98	0.96, 1.00	0.043	0.98	0.96, 1.00	0.071
Seniority (per year)	1.01	1.00, 1.03	0.128	1.03	1.00, 1.05	0.020	1.03	1.01, 1.05	0.010
Espace Mitteland	Ref.	Ref.	Ref.	Ref.	Ref.	Ref.	Ref.	Ref.	Ref.
Northwestern Switzerland	0.73	0.42, 1.27		0.52	0.25, 1.06		0.52	0.25, 1.09	
Eastern Switzerland	1.05	0.6, 1.83		1.23	0.60, 2.55		1.04	0.49, 2.23	
Lake Geneva Region	1.73	1.21, 2.47	0.001	1.63	1.01, 2.61	0.029	1.82	1.11, 3.00	0.021
Ticino	1.16	0.74, 1.81		1.08	0.59, 1.99		1.23	0.65, 2.32	
Central Switzerland	0.74	0.46, 1.20		0.75	0.38, 1.48		0.78	0.38, 1.58	
Zurich	0.65	0.37, 1.14		0.51	0.23, 1.1		0.52	0.24, 1.15	
Union 1	Ref.	Ref.	Ref.	Ref.	Ref.	Ref.	Ref.	Ref.	Ref.
Union 2	1.06	0.76, 1.49	0.716	1.68	1.04, 2.69	0.032	1.49	0.91, 2.42	0.111
Union 3	0.85	0.59, 1.22	0.366	1.05	0.61, 1.8	0.866	1.09	0.62, 1.91	0.754
Mandatory School	Ref.	Ref.	Ref.	Ref.	Ref.	Ref.	Ref.	Ref.	Ref.
Secondary School	1.36	1.01, 1.85	0.044	1.41	0.95, 2.1	0.088	1.41	0.94, 2.14	0.100
Superior School	1.19	0.85, 1.67	0.319	1.46	0.91, 2.34	0.119	1.42	0.87, 2.34	0.163
Apprenticeship	0.84	0.61, 1.15	0.273	1.01	0.67, 1.53	0.951	0.92	0.60, 1.42	0.718
Full-time	Ref.	Ref.	Ref.	Ref.	Ref.	Ref.	Ref.	Ref.	Ref.
Part-time	0.64	0.44, 0.92	0.016	0.74	0.44, 1.22	0.236	0.89	0.53, 1.50	0.660
Stress	1.32	1.01, 1.72	0.040	0.56	0.38, 0.82	0.003	0.47	0.31, 0.70	<0.001
Anxiety	2.51	1.71, 3.67	<0.001	1.65	1.00, 2.73	0.050	1.69	1.00, 2.85	0.050
Irritability	1.45	1.10, 1.92	0.008	0.92	0.63, 1.35	0.665	0.86	0.58, 1.29	0.469
Headache	1.55	1.17, 2.06	0.002	1.08	0.73, 1.58	0.705	1.04	0.70, 1.54	0.861
Back pain	1.60	1.23, 2.08	<0.001	1.17	0.81, 1.68	0.404	1.14	0.78, 1.66	0.489
Shoulder and neck pain	1.86	1.43, 2.43	<0.001	1.45	1.00, 2.1	0.053	1.40	0.95, 2.07	0.090
Upper limp muscular pain	1.95	1.38, 2.74	<0.001	1.13	0.70, 1.82	0.610	1.23	0.75, 2.02	0.406
Lower limp muscular pain	1.53	1.09, 2.15	0.013	0.87	0.55, 1.4	0.574	0.82	0.51, 1.33	0.428
Stomach pain	1.48	1.01, 2.16	0.046	0.75	0.44, 1.27	0.282	0.70	0.41, 1.20	0.190
Fatigue	1.83	1.41, 2.39	<0.001	1.27	0.87, 1.85	0.223	1.27	0.86, 1.89	0.232
Sleep disorders	2.21	1.69, 2.89	<0.001	1.71	1.17, 2.5	0.005	1.59	1.07, 2.36	0.022
Accident	1.98	1.32, 2.97	0.001	2.16	1.27, 3.68	0.004	1.82	1.05, 3.17	0.034
Night shift	1.23	1.08, 1.39	0.002	0.91	0.76, 1.09	0.301	0.86	0.72, 1.04	0.121
Working day > 10 hours	1.83	1.54, 2.17	<0.001	1.29	0.99, 1.69	0.058	1.24	0.94, 1.63	0.131
Driving time > 4 hours	1.65	1.43, 1.91	<0.001	1.28	1.01, 1.62	0.044	1.26	0.99, 1.62	0.062
Aggressive customers	1.29	1.11, 1.49	0.001	1.05	0.81, 1.38	0.702	1.03	0.77, 1.36	0.860
Aggressiveness of other road users	1.32	1.13, 1.53	<0.001	1.18	0.88, 1.59	0.262	1.24	0.92, 1.69	0.163
Cyclist behavior	1.38	1.18, 1.62	<0.001	1.11	0.87, 1.41	0.394	1.07	0.84, 1.37	0.582
Long periods without access to toilets	1.36	1.17, 1.57	<0.001	1.06	0.85, 1.32	0.596	0.99	0.79, 1.25	0.965
Impact on health	2.70	2.04, 3.58	<0.001	-	-	-	1.82	1.14, 2.91	0.012
Shortening of rest phase	1.80	1.36, 2.37	<0.001	-	-	-	1.45	0.89, 2.35	0.134
Overtime	1.32	1.01, 1.73	0.044	-	-	-	1.12	0.68, 1.83	0.667
Increased workload	1.71	1.30, 2.25	<0.001	-	-	-	0.88	0.59, 1.31	0.524
Replacement on short notice	2.70	2.04, 3.58	<0.001	-	-	-	0.85	0.54, 1.32	0.467

OR: Odd ration, C.I.: confidence interval
* Univariate logistic regression model

** Logistic regression model adjusted for sex, age, driving seniority, region, union, education, and work rate, based on the DAG 3 without SARS-CoV-2 crisis impacts
*** logistic regression model adjusted for sex, age, driving seniority, region, union, education, and work rate, based on the DAG 3 with SARS-CoV-2 crisis impacts

Table. S. 4. Explanatory factors of driving accidents (2022 survey among unionized bus drivers, Switzerland) (Stratégie Energétique et Santé, Switzerland, 2022)

	Model 1 *			Model 2 **			Model 3 ***		
	OR	95% C.I.	p-value	OR	95% C.I.	p-value	OR	95% C.I.	p-value
Female sex	0.88	0.5, 1.55	0.659	0.64	0.29, 1.42	0.271	0.68	0.29, 1.58	0.368
Age (per year)	1.02	0.99, 1.04	0.149	1.02	0.98, 1.05	0.339	1.02	0.98, 1.05	0.342
Seniority (per year)	1.03	1.01, 1.05	<0.001	1.03	1, 1.06	0.071	1.03	1, 1.07	0.037
Espace Mitteland	Ref.	Ref.	Ref.	Ref.	Ref.	Ref.	Ref.	Ref.	Ref.
Northwestern Switzerland	1.28	0.62, 2.65		1.09	0.4, 2.95		1.21	0.43, 3.43	
Eastern Switzerland	0.81	0.35, 1.91		0.55	0.16, 1.82		0.49	0.14, 1.73	
Lake Geneva Region	0.58	0.33, 1.03	<0.001	0.33	0.15, 0.74	0.025	0.33	0.14, 0.78	0.006
Ticino	0.44	0.19, 1		0.42	0.14, 1.27		0.54	0.18, 1.67	
Central Switzerland	1.40	0.74, 2.64		1.98	0.85, 4.58		1.89	0.77, 4.63	
Zurich	2.80	1.49, 5.29		2.03	0.82, 5.04		2.15	0.83, 5.52	
Union 1	Ref.	Ref.	Ref.	Ref.	Ref.	Ref.	Ref.	Ref.	Ref.
Union 2	0.85	0.5, 1.45	0.559	0.65	0.3, 1.4	0.270	0.50	0.22, 1.14	0.099
Union 3	1.70	1.05, 2.75	0.032	0.67	0.3, 1.48	0.320	0.64	0.28, 1.47	0.290
Mandatory School	Ref.	Ref.	Ref.	Ref.	Ref.	Ref.	Ref.	Ref.	Ref.
Secondary School	0.81	0.52, 1.25	0.335	0.66	0.38, 1.16	0.149	0.73	0.4, 1.32	0.294
Superior School	0.72	0.43, 1.2	0.204	0.69	0.34, 1.43	0.318	0.68	0.31, 1.48	0.334
Apprenticeship	2.13	1.23, 3.7	0.007	3.00	1.42, 6.36	0.004	2.93	1.34, 6.41	0.007
Full-time	Ref.	Ref.	Ref.	Ref.	Ref.	Ref.	Ref.	Ref.	Ref.
Part-time	0.77	0.43, 1.37	0.380	0.90	0.42, 1.95	0.795	1.06	0.47, 2.39	0.885
Stress	1.49	1.02, 2.18	0.040	1.38	0.81, 2.35	0.241	1.12	0.63, 1.99	0.701
Anxiety	1.24	0.77, 2.02	0.376	0.53	0.25, 1.13	0.101	0.46	0.21, 1.02	0.055
Irritability	1.62	1.1, 2.39	0.014	1.03	0.6, 1.77	0.927	0.85	0.48, 1.51	0.580
Headache	1.47	0.99, 2.17	0.054	1.25	0.73, 2.14	0.419	1.03	0.58, 1.83	0.910
Back pain	1.85	1.25, 2.74	0.002	1.58	0.93, 2.71	0.093	1.73	0.99, 3.02	0.056
Shoulder and neck pain	1.80	1.2, 2.7	0.005	1.36	0.77, 2.41	0.285	1.39	0.77, 2.51	0.281
Upper limp muscular pain	1.72	1.12, 2.65	0.013	1.07	0.56, 2.05	0.833	1.07	0.54, 2.1	0.850
Lower limp muscular pain	1.73	1.12, 2.67	0.014	1.59	0.84, 3.03	0.156	1.75	0.9, 3.39	0.097
Stomach pain	1.78	1.1, 2.88	0.019	1.43	0.73, 2.8	0.292	1.40	0.69, 2.85	0.353
Fatigue	2.05	1.37, 3.05	<0.001	1.35	0.76, 2.38	0.304	1.49	0.82, 2.7	0.193
Sleep disorders	1.66	1.13, 2.45	0.009	0.88	0.51, 1.54	0.658	0.68	0.38, 1.23	0.203
Sick leave	1.98	1.32, 2.97	0.001	2.16	1.26, 3.72	0.005	2.06	1.16, 3.65	0.014
Night shift	1.02	0.85, 1.22	0.857	0.89	0.69, 1.14	0.347	0.88	0.67, 1.14	0.328
Working day > 10 hours	1.52	1.15, 2	0.003	1.18	0.79, 1.74	0.419	1.08	0.71, 1.63	0.714
Driving time > 4 hours	1.31	1.05, 1.62	0.014	0.90	0.63, 1.28	0.547	0.84	0.58, 1.23	0.377
Aggressive customers	1.24	1, 1.53	0.050	0.95	0.64, 1.42	0.819	0.94	0.62, 1.45	0.789
Aggressiveness of other road users	1.28	1.02, 1.6	0.036	0.83	0.54, 1.29	0.413	0.81	0.51, 1.29	0.370
Cyclist behavior	1.29	1, 1.65	0.048	1.21	0.85, 1.74	0.291	1.21	0.83, 1.76	0.328
Long periods without access to toilets	1.51	1.18, 1.92	0.001	1.36	0.98, 1.89	0.070	1.21	0.86, 1.71	0.279
Urban service	Ref.	Ref.	Ref.	Ref.	Ref.	Ref.	Ref.	Ref.	Ref.
Mixed service	0.82	0.53, 1.26	0.363	0.53	0.28, 0.99	0.047	0.52	0.27, 1.01	0.052
Regional service	0.66	0.38, 1.16	0.145	0.37	0.16, 0.88	0.025	0.29	0.11, 0.73	0.009
Impact on health	2.95	1.94, 4.47	<0.001	-	-	-	3.05	1.51, 6.14	0.002
Shortening of rest phase	1.66	1.12, 2.47	0.012	-	-	-	0.96	0.47, 1.97	0.905
Overtime	1.65	1.11, 2.45	0.012	-	-	-	1.68	0.87, 3.25	0.123

Increased workload	1.76	1.19, 2.62	0.005	-	-	-	0.80	0.39, 1.66	0.554
Replacement on short notice	2.95	1.94, 4.47	<0.001	-	-	-	0.63	0.35, 1.13	0.119

OR: Odd ration, C.I.: confidence interval

* Univariate logistic regression model

** Logistic regression model adjusted for sex, age, driving seniority, region, union, education, and work rate, based on the DAG 4 without SARS-CoV-2 crisis impacts

*** logistic regression model adjusted for sex, age, driving seniority, region, union, education, and work rate, based on the DAG 4 with SARS-CoV-2 crisis impacts
